# Exposure-response analyses of efzofitimod in patients with pulmonary sarcoidosis

**DOI:** 10.3389/fphar.2023.1258236

**Published:** 2023-10-03

**Authors:** Gennyne Walker, Ryan Adams, Lauren Guy, Abhijeeth Chandrasekaran, Nelson Kinnersley, Pavithra Ramesh, Lu Zhang, Fran Brown, Vis Niranjan

**Affiliations:** ^1^ aTyr Pharma, San Diego, CA, United States; ^2^ RxMD, Chennai, India; ^3^ Octa Consulting Services Ltd., Harpenden, United Kingdom; ^4^ Certara Inc., Princeton, NJ, United States

**Keywords:** efzofitimod, pulmonary sarcoidosis treatment, exposure-response relationship, steroid-sparing effect, forced vital capacity, King’s Sarcoidosis Questionnaire-Lung

## Abstract

**Background:** Preliminary evidence for efficacy in pulmonary sarcoidosis has been shown for efzofitimod. Here we present supportive evidence of efficacy based on an exposure-response analysis.

**Methods:** Data from two studies (Phase 1, N = 24, single dose in healthy volunteers, and Phase 1b/2a, N = 25, multiple doses over 24 weeks in participants with pulmonary sarcoidosis) were used to build a population pharmacokinetic model. Using this model, the relationship between efzofitimod exposure and three prespecified efficacy parameters [mean daily oral corticosteroid (OCS) dose, percent-predicted forced vital capacity (ppFVC) and King’s Sarcoidosis Questionnaire-Lung (KSQ-Lung) score] was explored. Linear regression described the relationship of efzofitimod exposure and OCS reduction, ppFVC and KSQ-Lung score. Logistic regression related efzofitimod exposure to the probability of achieving a minimal clinically important difference for ppFVC and KSQ-Lung score. Due to the small study size, trends (not statistical significance) in relationships are reported.

**Results:** In patients with pulmonary sarcoidosis, as efzofitimod exposure increased, the mean daily OCS dose decreased, and ppFVC and KSQ-Lung score improved over baseline. The slope for all the endpoints by both linear and logistic regression showed an improving trend with increased exposure.

**Conclusion:** These preliminary findings of a positive exposure-response across multiple efficacy endpoints support the claim that proof of concept has been established for the use of efzofitimod in pulmonary sarcoidosis.

**Clinical Trial Registration:** clinicaltrials.gov, identifier NCT03824392

## 1 Introduction

Efzofitimod is a novel immunomodulatory Fc fusion protein in development for the treatment of pulmonary sarcoidosis (PS). Sarcoidosis is a multisystem, immune-mediated granulomatous disorder that affects the lungs in more than 90% of patients, resulting in interstitial lung disease (ILD) (Culver and Judson., 2019; Baughman et al., 2011). Around one in ten patients will die from the disease within 10 years (Kirkil et al., 2017), with pulmonary disease being among the most common reasons for death (Swigris et al., 2011).

No new drugs have been approved by the United States (US) Food and Drug Administration (FDA) for sarcoidosis since the corticotropin injection in 1952 (prior to current health authority guidelines). Oral corticosteroids (OCS), especially prednisone, are the first line of treatment for PS (Baughman et al., 2021a). Since sarcoidosis is often diagnosed in early adulthood, many patients remain on OCS for years. The toxicity of cumulative glucocorticoid exposure (dose × duration) has been well recognized in many diseases, and sarcoidosis is no exception (Khan et al., 2017; Judson et al., 2015). Long-term corticosteroid use is associated with significant side effects including substantial weight gain, development of insulin resistance, osteoporosis, risk of infection, and overall reduced quality of life. Guidelines from the European Respiratory Society (ERS) recommend steroid reduction as a critical treatment outcome measure in the management of PS (Baughman et al., 2021a). ERS guidelines also consider forced vital capacity (FVC) as an important outcome measure (Baughman et al., 2021a). In small observational studies in steroid-naïve patients with PS, FVC improves in response to OCS treatment. Likewise, in patients with worsening PS while on steroids, FVC improves in response to an increase in steroid dose (Broos et al., 2018; McKinzie et al., 2010). Notwithstanding the steroid toxicity, this responsiveness underpins the use of OCS as first-line therapy. Given the above, the ability to taper OCS without a worsening in FVC and pulmonary symptoms (i.e., cough, dyspnea) represents an important metric for evaluating any new agent.

Cytotoxic immunomodulators such as methotrexate are recommended as second-line therapy when patients experience progressive disease or cannot tolerate their OCS therapy. Biologic immunomodulators (e.g., infliximab and adalimumab) are recommended for continued disease or patients who relapse while on treatment with immunomodulators. Subsequent lines of therapy include treatment with rituximab, Janus kinase inhibitors, or repository corticotropin injection (Acthar Gel) on a case-by-case basis (Baughman et al., 2021a). ERS recommendations on these drugs are qualified as having minimal supportive evidence (Baughman et al., 2021a).

Despite the significant unmet need, over the last decade there have been few well designed, adequately controlled interventional studies in PS. Efzofitimod is the first development candidate to enter a confirmatory Phase 3 study in PS [ClinicalTrials.gov, Identifier NCT05415137, 2022; Obi et al., 2022].

Efzofitimod was evaluated in both a Phase 1 and a Phase1b/2a study (both double blind, placebo controlled) and was found to be safe and well-tolerated at all doses tested up to 5 mg/kg. In the Phase 1b/2a study, dose-dependent trends in pre-specified efficacy endpoints compared to placebo were observed across several parameters: steroid-sparing effect (SSE), lung function, and patient-reported outcomes (PROs) (Culver et al., 2022). Here, we present the findings of an exposure-response (E-R) analysis from this Phase 1b/2a study. In an E-R analysis, the relationship between amount of drug exposure [pharmacokinetic (PK) parameters] and the subsequent treatment effect is explored. However, unlike in healthy volunteer studies, a study in a patient population precludes the frequent blood sampling that is required to calculate the exposure parameters by simple methods. In this analysis, we therefore apply a population PK (PPK) model approach to derive exposure parameters.

In PPK modeling, data from participants who have frequent blood sampling (e.g., healthy volunteers from a Phase 1 study) and a well-defined concentration-time curve (profile) are combined with data from participants (e.g., PS patients from the Phase 1b/2a study) who have sparse blood sampling (not enough to characterize the curve on their own). PPK modeling leverages the rich data from healthy volunteers to “fill in” spaces in the sparse PK profiles of patients. The model is tested by comparing how the observed concentrations fall within the model predictions. A model that describes the data well can be used to support future studies through exposure predictions and dosing simulations.

An E-R analysis is an accepted approach for providing clinical evidence of efficacy in early development and dose exploration, including proof of concept (PoC) (US FDA, 2003).

## 2 Materials and methods

### 2.1 Studies included

Data from the first-in-human, Phase 1, single ascending dose study (ATYR1923-C-001) of efzofitimod in healthy volunteers and data from a Phase 1b/2a, multiple ascending dose study (ATYR1923-C-002) of efzofitimod in participants with PS were included in the analysis (Table 1). Both studies included multiple sequential cohorts. Within each cohort, participants were randomized 1:2 to placebo or efzofitimod.

**TABLE 1:** Summary of studies contributing data to the analyses.

	Cohorts/Dose (mg/kg)	Placebo (n)	Efzofitimod (n)
Phase 1—healthy volunteers/single i.v. infusion/sequential cohorts	0.03	2	4
	0.1	2	4
	0.3	2	4
	1	2	4
	3	2	5^a^
	5	2	4
Randomized (n)		12	25
PK-evaluable (n)		0	24^a^
Phase 1b/2a—patients with PS/six Q4W i.v. infusions/sequential cohorts	1	4	8
	3	4	8
	5	4	9^b^
Randomized and dosed (n)		12	25
PK-evaluable (n)		0	25
Efficacy-evaluable (n)		11^c^	21^c^

i.v., intravenous; PK, pharmacokinetic; PS, pulmonary sarcoidosis; Q4W, once every 4 weeks.

^a^ A fifth participant was added to the 3 mg/kg cohort to replace a participant in whom the infusion was not completed.

^b^ An extra participant was randomized in the 5 mg/kg cohort to replace a participant who had received only one dose.

^c^ All participants who had received at least four doses of study drug.

In Phase 1, 37 participants were randomized (across all cohorts) to placebo (*n* = 12) or efzofitimod (*n* = 25). Blood samples for the analysis of serum efzofitimod concentrations were collected predose, at 0.5 h after the start of infusion, at 1 h (just prior to the end of infusion), and at 2, 4, 8, 12, 18, 24, 48, 96, 168, 336, 504, and 672 h. Concentration data from participants (N = 24) who received one dose of efzofitimod at 0.03, 0.1, 0.3, 1.0, 3.0, and 5.0 mg/kg with at least one valid post-dose serum concentration were included in the PPK analysis (PK-evaluable population). One of the 25 enrolled participants, in whom the infusion was not completed, was excluded from the PK-evaluable population.

In Phase 1b/2a, 37 participants were randomized (across all cohorts) to placebo (*n* = 12) or efzofitimod (*n* = 25). Efzofitimod was prescribed every 4 weeks for six doses. Blood samples for the analysis of serum efzofitimod concentrations were collected pre-dose on all six dosing days. Additional postdose samples were collected on Day 1 (first dose) and Week 20 (last dose) at 1 h (just prior to the end of infusion) and between 4 and 6 h after the start of infusion. Concentration data from participants on active treatment (N = 25) who received at least one dose of efzofitimod 1.0, 3.0 or 5.0 mg/kg every 4 weeks and with at least one valid postdose serum concentration, were included in the PPK analysis (PK-evaluable population).

Participants who received at least four doses of efzofitimod or placebo were included in the E-R analysis (efficacy-evaluable population; Table 1). There were 32 participants who received four or more doses of placebo or efzofitimod: six doses (9 placebo, 17 efzofitimod), five doses (0 placebo, 2 efzofitimod), four doses (2 placebo, 2 efzofitimod). Serum samples from both studies were analyzed for efzofitimod concentration by a validated immunoassay.

The Phase 1 study was conducted under a Clinical Trial Notification, submitted to the Australian Therapeutic Goods Administration. The Phase 1b/2a study was conducted under a US Investigational New Drug Application. Both studies were conducted in accordance with the Declaration of Helsinki and with approval of the appropriate local ethics committees. Written informed consent was obtained from all participants before study entry.

The studies contributing to the analyses are shown in Table 1. The PK-evaluable population was used to build the PPK model, and the efficacy-evaluable population was used to perform the E-R analysis.

### 2.2 PPK model development

PPK dataset construction, exploratory plots, statistical summaries, data exploration, E-R modeling and simulations were performed in R (Version 4.0) (R Core Team, 2022). PPK model development was conducted using the first-order conditional estimation with interaction in NONMEM^®^ (Version 7.4; ICON Development Solutions; Hanover, MD, United States).

Following structural model selection, covariate screening was performed via stepwise forward addition and backward elimination. Covariates tested included baseline body weight, age, sex, race, ethnicity, baseline albumin on clearance (CL) and volume [central volume of distribution (V1) and peripheral volumes of distribution (V2, V3)], and the effect of baseline liver function (alkaline phosphatase (ALP), alanine transaminase (ALT), aspartate transaminase (AST), and bilirubin) and renal function [serum creatinine, estimated glomerular filtration rate (eGFR), and creatinine CL (CrCL)] on efzofitimod CL. Model selection was guided by standard goodness-of-fit metrics.

### 2.3 Use of PPK model to generate individual predicted exposure (time-averaged AUC) estimates

The PPK model was used to calculate CL and volume PK parameters for each participant in the model dataset. These individual parameter estimates were then used to calculate drug exposures over time such as the area under the concentration-time curve (AUC) for each participant.

Of the several individual exposure (PK) parameters that can be derived from the PPK model, AUC was used as the exposure parameter of interest as it best represents exposure to a therapy over a prolonged period. In chronic therapy, AUC is typically the most highly correlated PK parameter to efficacy. Time-averaged AUCs of efzofitimod were calculated using each participant’s time-averaged cumulative efzofitimod exposure (AUC) as follows:

$$Time\;averaged\;AUC=\frac{Total\;AUC\;in\;study\;duration}{Study\;duration}$$

Use of the time-averaged AUC allows for the calculation of an average exposure and for the comparison of exposures following different numbers of doses to different participants (see Table 2). It was confirmed there was a good correlation between time-averaged AUC and dose.

**TABLE 2:** Exposure-response simulation dose regimen scenarios.

Scenario	Regimen	Dosage	Notes
1		Placebo	—
2	Weight-based	3 mg/kg every 28 days for 6 cycles	—
3	Weight-based	5 mg/kg every 28 days for 6 cycles	—
4	Flat dosing	450 mg every 28 days for 6 cycles	450 mg = 5 mg/kg × 90 kg^a^
5	Loading dose	900 mg loading dose on Day 1 + 4 mg/kg every 28 days for 5 doses	Equivalent to 5 mg/kg for a 90 kg^a^ participant
6	Weight-based	7 mg/kg every 28 days for 6 cycles	—

^a^ 90 kg was the median weight in the Phase1b/2a study.

### 2.4 Clinical response parameters from the phase 1b/2a study

In the Phase 1b/2a study, efficacy parameters were evaluated in three areas: SSE, lung function, and PROs.

Starting on Day 15 of this 24-week study, the investigator initiated a protocol-guided taper (reduction) in OCS for each participant from a starting dose of 10–25 mg/day of prednisone (or equivalent) to a target dose of 5 mg/day, to be completed on or before Day 50. The OCS dose was to be tapered by 5 mg/day every 1–2 weeks, depending on the starting dose. Rescue OCS was allowed if the participant had worsening cough/dyspnea per the investigator’s clinical judgment. While Day 1 to Week 24 was considered the treatment period, Day 51 to Week 24 was considered the post-taper period.

OCS reduction was analyzed as defined in the study protocol, using mean daily OCS dose over the post-taper period. The mean was calculated using cumulative OCS dose divided by the duration (in days) over which the cumulative OCS was recorded. Lung function was monitored with percent-predicted forced vital capacity (ppFVC), the most clinically meaningful parameter in ILDs. The King’s Sarcoidosis Questionnaire-Lung (KSQ-Lung) score was included in the E-R analysis as this is the most representative PRO for PS (scoring based on cough, dyspnea, and chest pain) (Baughman et al., 2021a). There were thus three efficacy parameters assessed for an E-R relationship: OCS reduction, ppFVC and KSQ-Lung score.

In this E-R analysis, there were two sets of endpoints used for the efficacy parameters. The first set of endpoints (protocol prespecified) evaluated the percent change from baseline for the mean daily OCS dose in the post-taper period, ppFVC at Week 24, and KSQ-Lung score at Week 24.

A second set of endpoints was used in responder analyses based on a *post hoc* definition of a threshold (minimal clinically important difference [MCID]). The *post hoc* responder analyses were undertaken for ppFVC and KSQ-Lung score. A responder analysis for OCS dose was not applicable as no specific thresholds for a meaningful response were identified in the literature. For ppFVC, MCID varied depending on the indication, with no specific threshold for PS. In another ILD (systemic sclerosis associated ILD), a 2.5% change at Week 24 was considered to be an MCID (Khanna et al., 2009). For KSQ-Lung score, a ≥four-point change is a validated MCID for sarcoidosis (Baughman et al., 2021b).

### 2.5 E-R analysis

The E-R analysis was undertaken for participants in the efficacy-evaluable population from the Phase 1b/2a study (Table 1). In this study, the time-averaged AUC (exposure) tertiles of the efficacy-evaluable population were used in the exploratory analysis and modeling. The AUCs for efzofitimod were averaged for two intervals: treatment period (Day 1 to Week 24) and the post-taper period (Day 51 to Week 24).

E-R relationships between continuous endpoints (percent change from baseline) and AUC were explored and modeled using a linear regression. For the responder analyses, a logistic model with a linear relationship to time-averaged AUC was used to explore the E-R relationship. Given the exploratory nature of the Phase 1b/2a study, formal (statistical) hypothesis testing would be expected to be underpowered, so confidence and prediction intervals have been included to convey uncertainty of point estimates (Amrhein et al., 2019).

The impact on E-R of demographic covariates (e.g., age, sex, race, and body weight), baseline disease characteristics (e.g., duration of disease and concomitant immunomodulator), and efficacy endpoints at baseline (i.e., mean daily OCS dose, ppFVC, KSQ-Lung score) was assessed using a forward univariate search, incorporating all significant covariates into a full model, followed by a backward elimination.

**FIGURE 1:**
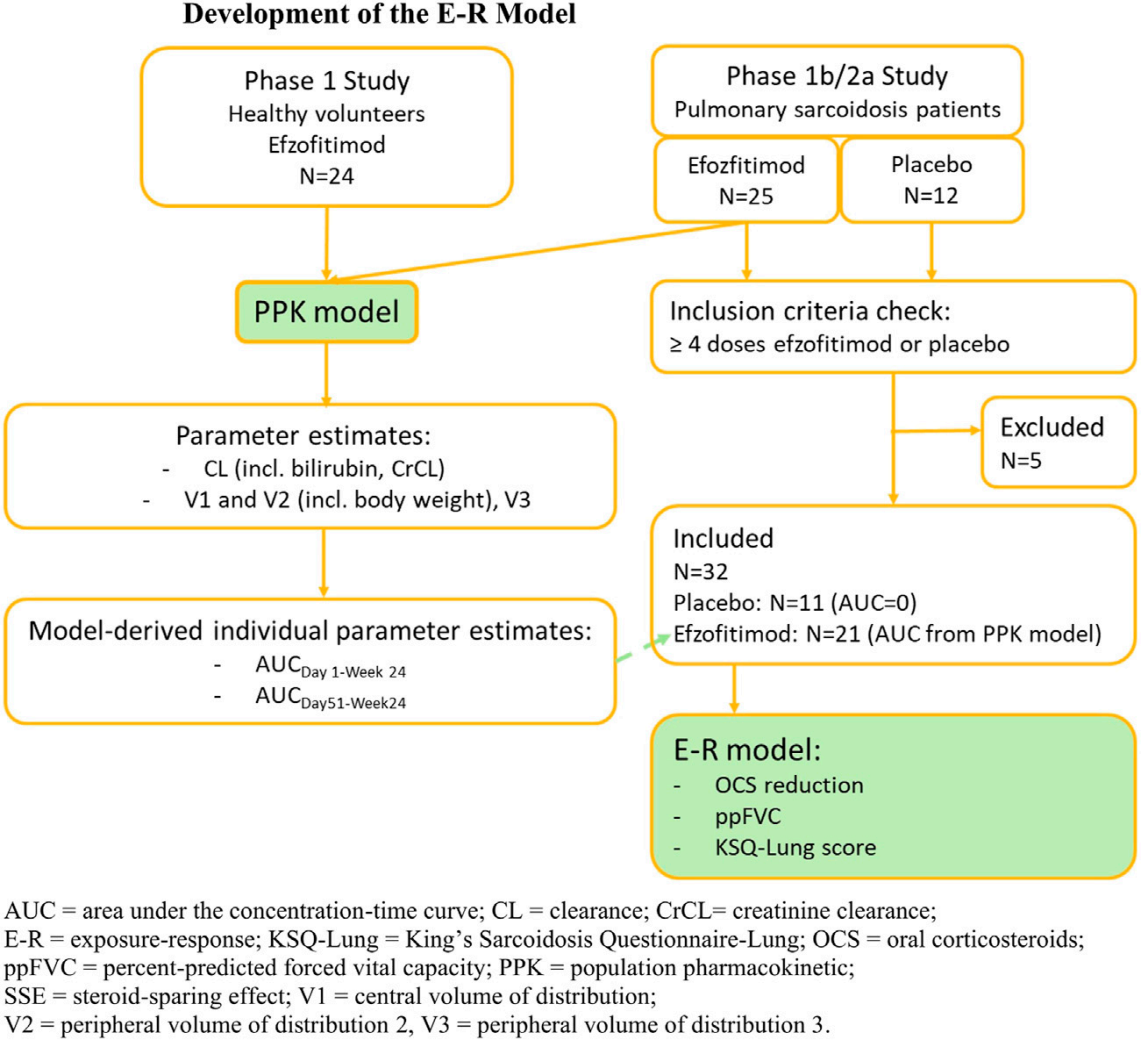
Development of the E-R model.

The study flow for E-R analysis is depicted in Figure 1.

### 2.6 Evaluation of dosing for future studies

The models developed (PPK and E-R) were used to support dose selection for future studies and to compare weight-based dosing, flat dosing, and loading-dose regimens. The PPK model was used to simulate expected exposures for the dose regimens listed in Table 2. The logistic E-R models developed for ppFVC and KSQ-Lung score were used to simulate the probability of response for these regimens using the simulated exposures.

## 3 Results

### 3.1 Description of datasets

In the PPK analysis, a total of 49 participants were included in the dataset used for the PPK model development, representing a total of 565 concentration samples. Demographic characteristics are shown in Table 3. The Phase 1 study was conducted in healthy volunteers in Australia while the Phase 1b/2a study was conducted in patients with PS in the US. In the PK-evaluable population, there were thus expected differences in the populations between the study in healthy volunteers (*n* = 24) and PS patients (*n* = 25). Participants in the Phase 1 study were generally younger than those in the PS population (mean 24 versus 52 years, respectively). The mean body weight of healthy volunteers was lower than the mean body weight of the PS population (74 versus 98 kg) and healthy volunteers had lower variability in bodyweight than the PS population did (standard deviations of 10.5 versus 25.2 kg). There were more Black or African American participants in the PS population (*n* = 11) compared to healthy volunteers (*n* = 0). There were five Asian healthy volunteers (study in Australia), and none in the PS population. The effect of demographics is discussed in Section 3.2.

**TABLE 3:** Demographics—PK-evaluable and efficacy-evaluable populations.

	PK-evaluable		Efficacy-evaluable (phase 1b/2a)				
Characteristics	Phase 1 (n = 24)	Phase 1b/2a (n = 25)	Placebo (n = 11)	Efzofitimod			
				Tertiles^a^			Total (n = 21)
				[805; 3,638] (n = 7)	[3,638; 6,198] (n = 7)	[6,198; 14,860] (n = 7)	
Age (years), mean (SD)	23.8 (3.94)	52.3 (10.3)	53.2 (10.4)	55.7 (11.6)	51.0 (10.8)	49.9 (10.5)	52.2 (10.7)
Gender, n							
Male	11	12	4	4	4	3	11
Female	13	13	7	3	3	4	10
Body weight (kg), mean (SD)	74 (10.5)	97.6 (25.2)	89 (14.15)	87 (7.94)	92.5 (21.9)	116 (35.7)	98.5 (26.7)
Race, n							
White	16	14	8	4	5	3	12
Black or African American	0	11	3	3	2	4	9
Asian	5	0	0	0	0	0	0
Other	3	0	0	0	0	0	0
Ethnicity, n							
Not Hispanic/Latino	22	24	10	7	7	7	21
Hispanic/Latino	2	1	0	0	0	0	0

AUC, area under the concentration-time curve; PK, pharmacokinetic; SD, standard deviation.

^a^ Tertiles are calculated by AUC, from Day 1 to Week 24.

In the E-R analysis, only participants from the Phase 1b/2a study were included in the efficacy-evaluable population. In this study, the time-averaged AUC (exposure) tertiles were used in the exploratory analysis and modeling. Participant demographic and baseline characteristics are summarized by tertiles (*n* = 7 for each tertile) of time-averaged AUC from Day 1 to Week 24 in Tables 3, 4, respectively.

**TABLE 4:** Baseline disease characteristics—efficacy-evaluable population (Phase 1b/2a Study).

Characteristics	Placebo (n = 11)	Efzofitimod			
		Tertiles^a^			Total n = 21
		[805; 3,638] (n = 7)	[3,638; 6,198] (n = 7)	[6,198; 14,860] (n = 7)	
Duration of disease (years), median (range)	2.83 (0.5, 8.4)	4.41 (1.5, 19.6)	3.48 (0.6, 15)	1.89 (0.5, 28)	3.55 (0.5, 28)
Immunomodulator Use, n	6	2	1	3	6
Prednisone equivalent dose^b^ (mg/day), mean (SD)	13.6 (4.5)	11.4 (3.78)	13.6 (6.27)	14.3 (3.45)	13.1 (4.6)
ppFVC, mean (SD)	78.1 (11.7)	66.3 (8.54)	84.6 (8.32)	83.4 (18.8)	78.1 (14.9)
KSQ-Lung score, mean (SD)	42.4 (10.6)	50.6 (11.4)	53.8 (13.3)	48.3 (15.3)	50.9 (13)

AUC, area under the concentration-time curve; FVC, forced vital capacity; KSQ, king sarcoidosis questionnaire; n = number of participants with the measure; OCS, oral corticosteroids; ppFVC, percent-predicted forced vital capacity; SD, standard deviation.

Baseline measurements were defined as the last measurement assessed on or before the first dose date. If multiple measurements were taken on Day 1, the last measurement before the first dose was used as baseline.

^a^ Tertiles are calculated by AUC, from Day 1 to Week 24.

^b^ Any OCS, that is not prednisone was converted to a prednisone-equivalent OCS.

Participants on placebo (*n* = 11) were added for reference. There were some imbalances, consistent with the small number of participants evaluated. There were fewer African Americans in the placebo arm (3/11) compared to the highest tertile (4/7). Interestingly, weight increased from the low to the high tertiles (Table 3). This speaks to the potential suitability for fixed dosing, discussed in Section 3.4. In the context of small sample sizes and the large ranges, there were no apparent differences in baseline disease characteristics (Table 4).

### 3.2 PPK model

**FIGURE 2:**
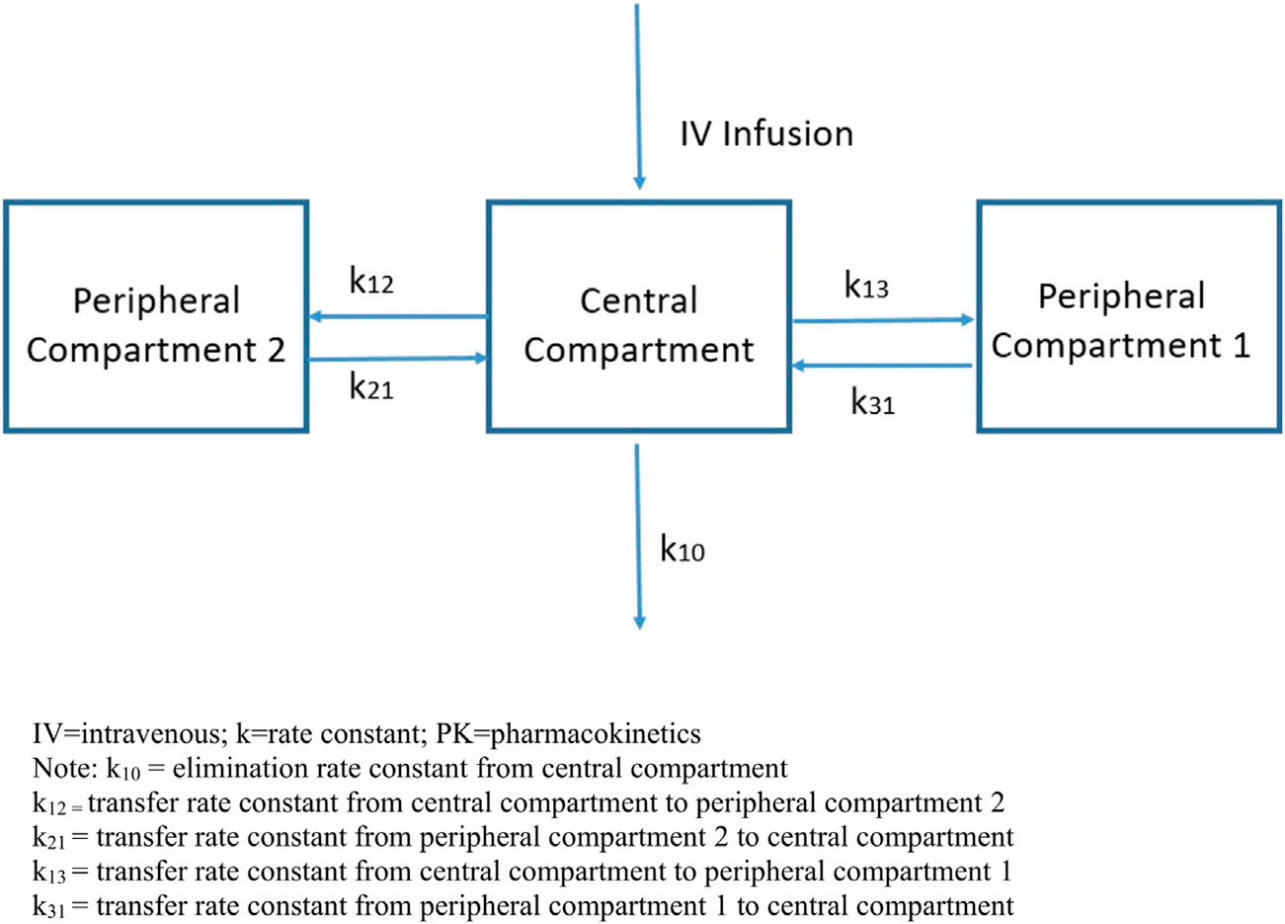
Three-compartment PK model.

A three-compartment model provided the best description of the PK data. These included a central compartment and two peripheral compartments with first-order elimination from the central compartment (Figure 2).

**FIGURE 3:**
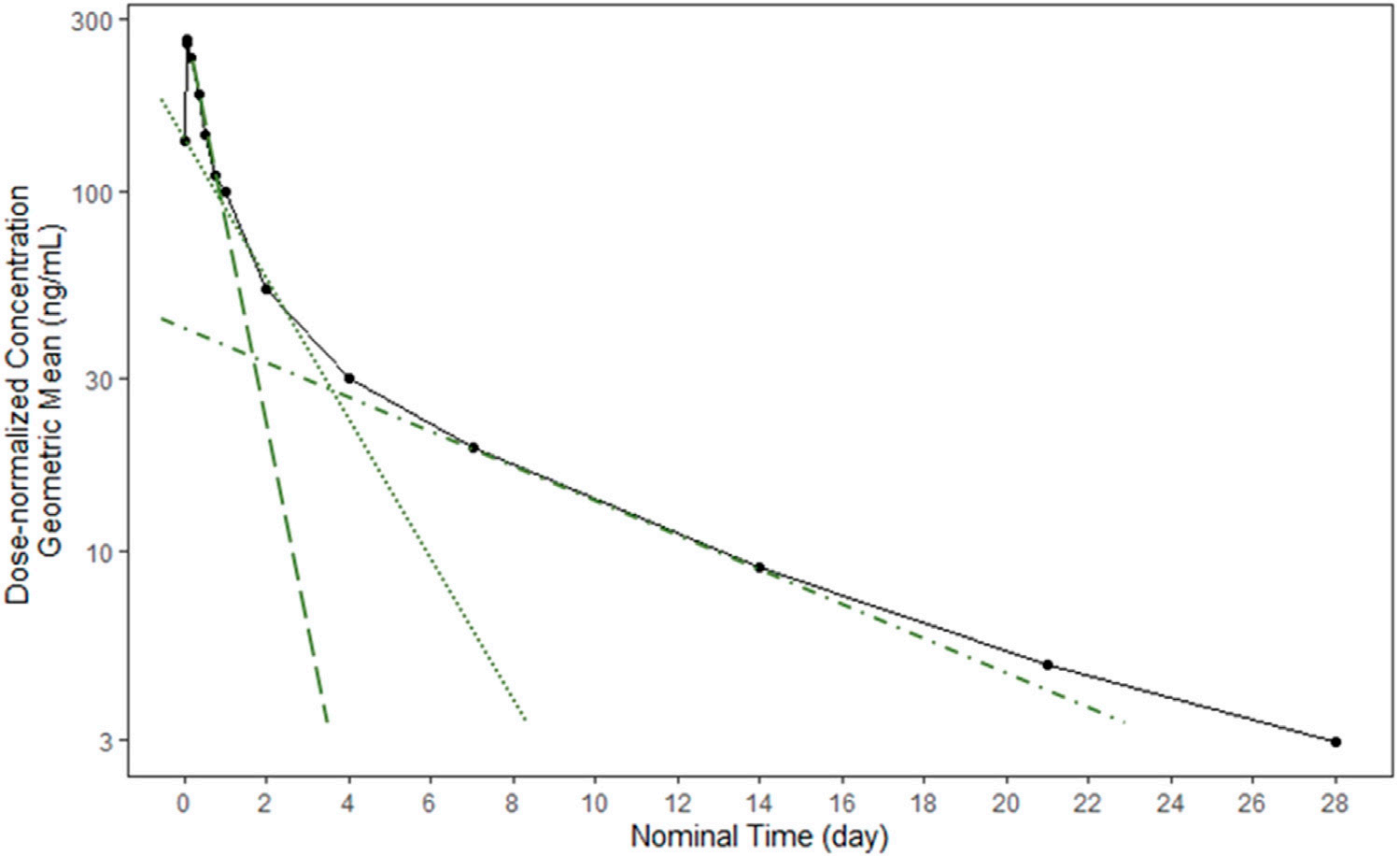
Mean efzofitimod concentration-time profile.

The mean concentration-time profile for efzofitimod (solid black line) is displayed in Figure 3.

Following i.v. infusion of efzofitimod, three distinct elimination phases were apparent in the concentration-time profiles. Each phase can be described by calculating the slope of the line in each of the three regions (green lines).

Compartments used in mathematical models do not have direct physical correlates (i.e., they are not physiologic spaces); they indicate the relative dynamics of how a compound behaves in the body. The model included terms which described the CL of compound from the body as well as parameters which described the rates at which it moved in and out of the identified compartments.

The estimated population central value of efzofitimod CL was 1.68 L/day, consistent with CL values of fusion proteins of similar size reported in the literature (Khanna et al., 2009; Pérez-Ruixo et al., 2009). Population central estimates of volumes of distribution (Vd) for the central compartment (V1) and two peripheral compartments (V2, V3) were 3.94, 3.3, and 7.43 L, respectively, constituting a total Vd of approximately 15 L, and indicative of distribution that is primarily limited to the extracellular volume. The apparent Vd for fusion proteins is usually small and is limited to the volume of the extracellular space, due to low membrane permeability resulting from their large size and hydrophilicity.

The overall estimated half-life of efzofitimod accounting for all phases of elimination was 11.3 days, and the terminal elimination half-life was 9 days.

Race, sex, and age were not significant covariates in the PPK model for efzofitimod. Bodyweight, CrCL and bilirubin were identified as significant covariates for the PPK model but were calculated to have a small impact on efzofitimod exposure and were regarded as not clinically meaningful.

Body weight was a statistically significant covariate on efzofitimod Vd (V1 and V2). Participants with heavier weight are expected to have a higher Vd. Across body weights encountered in this study, efzofitimod exposure (AUC) is predicted to increase by 30% at the 90th percentile of body weight and decrease by 19% at the 10th percentile of body weight (relative to the median value of 82 kg) over the weight range studied (55–157 kg).

Baseline CrCL and total bilirubin levels were statistically significant covariates on efzofitimod CL. When CrCL ranged from 103 mL/min to 187 mL/min (10th to 90th percentiles), steady-state AUC varied from 10% to −10% compared to a typical participant. Impaired renal function is known to decrease the renal CL of therapeutic proteins/peptides with molecular weights <69 kDa (US FDA, 2020), with the more significant effects for molecules <50 kDa (EMA, 2007). The molecular weight of efzofitimod is 64.5 kDa; therefore, the modest impact of CrCL on efzofitimod CL is in line with previous data for therapeutic proteins and suggests that renal function may have a limited role in efzofitimod elimination. As bilirubin varied from 3.2 μmol/L to 19.6 μmol/L (10th to 90th percentiles), the steady-state AUC varied from −8% to 10% compared to a typical participant. These findings are consistent with the fact that all study participants included in this analysis had total bilirubin and CrCL within the normal range. Other markers of hepatic function (albumin and hepatocellular and canalicular enzyme serum levels) were not identified as significant covariates on efzofitimod CL.

The PK parameters and covariates for the final PPK model are shown in Supplementary Table S1.

### 3.3 E-R model

Only data from the Phase 1b/2a study in participants with PS was included in the E-R models. The E-R analysis is presented initially for the protocol prespecified endpoints evaluating percent change from baseline and subsequently for the *post hoc* endpoints evaluating a responder analysis. The equations for each of these E-R analysis are presented in Supplementary Equations S1. The exposure parameter for all these analyses, with the exception of that for OCS reduction, was the time-averaged AUC from Day 1 to Week 24. As the OCS reduction endpoint was the mean daily dose of steroids in the post-taper period, the exposure parameter was the time-averaged AUC from Day 51 to Week 24.

Only the baseline values of the respective efficacy parameters were significant covariates and were retained in the E-R model; no other covariates were significant for the analysis.

**FIGURE 4:**
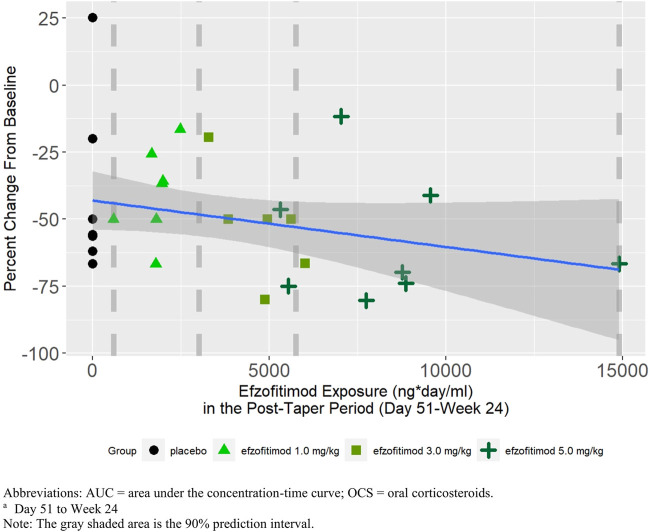
Mean daily OCS dose post-taper period^a^—percent change from baseline.

A negative slope was observed in the linear regression between percent change from baseline in mean daily OCS dose post-taper and exposure (time-averaged AUC from Day 51 to Week 24), indicating greater OCS reduction was achieved as exposure increased (Figure 4).

**FIGURE 5:**
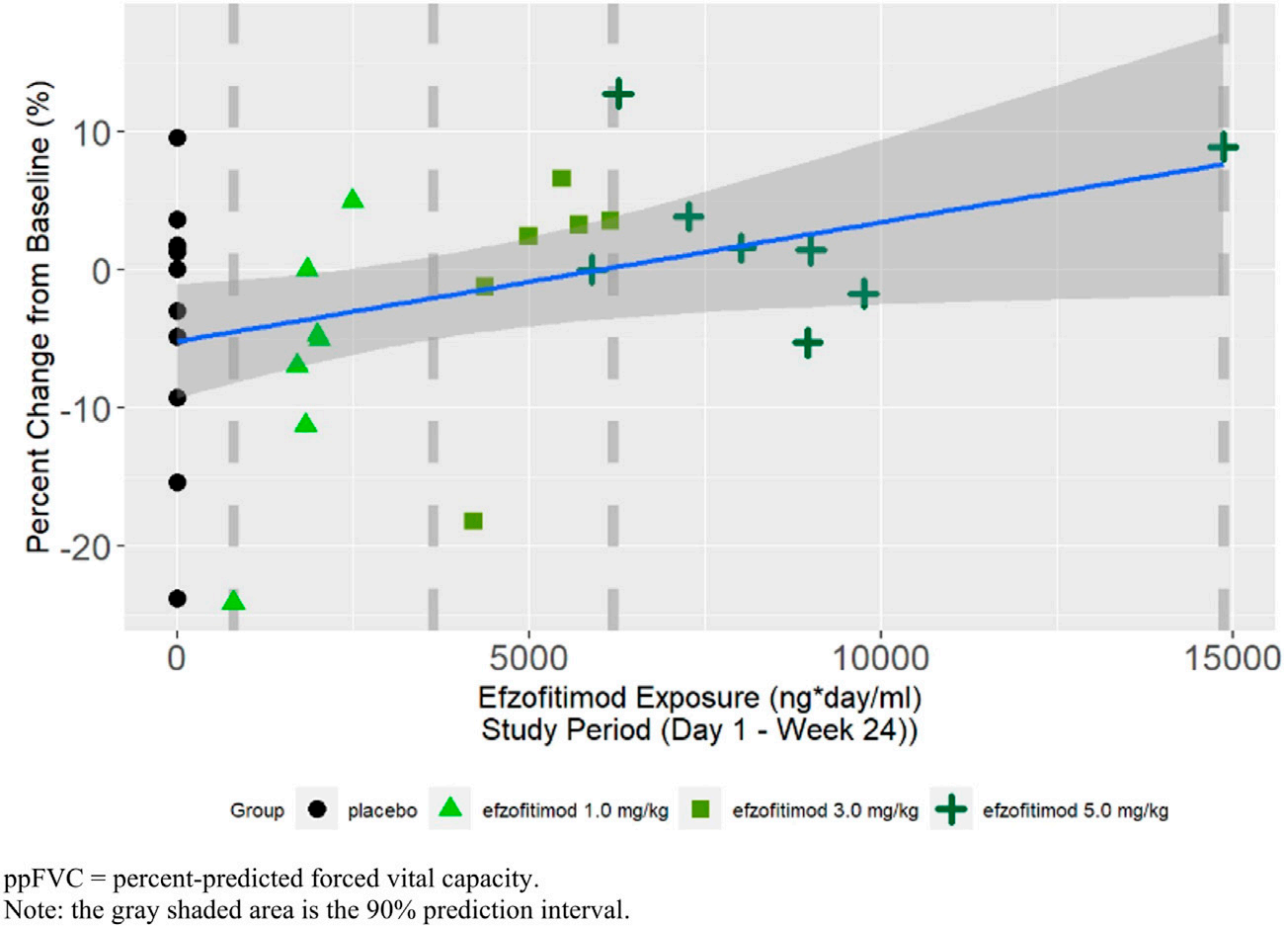
ppFVC at Week 24—percent change from baseline.

A positive slope was observed in the linear regression between percent change from baseline in ppFVC at Week 24 and efzofitimod exposure, indicating greater increases in ppFVC with increasing exposure (Figure 5).

**FIGURE 6:**
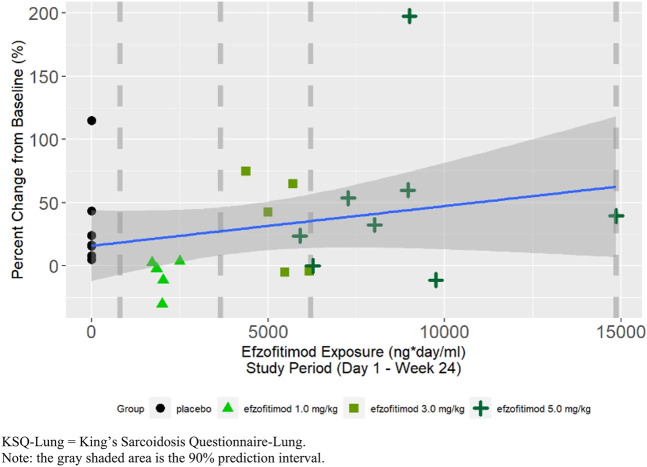
KSQ-lung score at week 24—percent change from baseline.

A positive slope was observed in the linear regression between percent change from baseline in KSQ-Lung score at Week 24 and efzofitimod exposure, indicating improvement in KSQ-Lung scores with increasing exposure (Figure 6).

**FIGURE 7:**
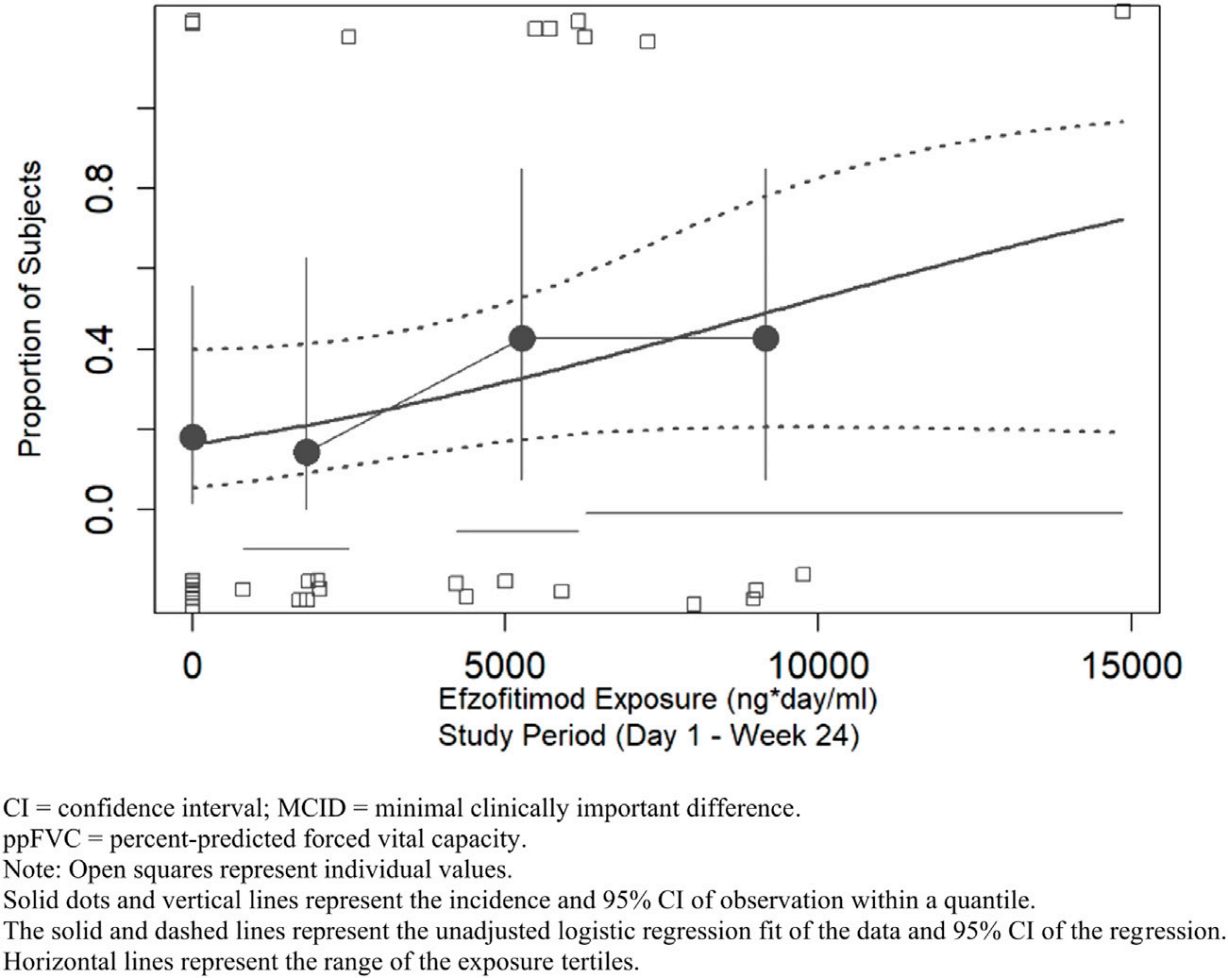
ppFVC—proportion of participants achieving MCID threshold.

A linear logistic regression analysis was performed for ppFVC to explore the relationship between efzofitimod exposure and the probability of achieving an MCID in ppFVC. Responders in this analysis were defined as participants who achieved a 2.5% increase in ppFVC (Khanna et al., 2009). This analysis supports the positive relationship between exposure and the probability of achieving the MCID in ppFVC (Figure 7).

**FIGURE 8:**
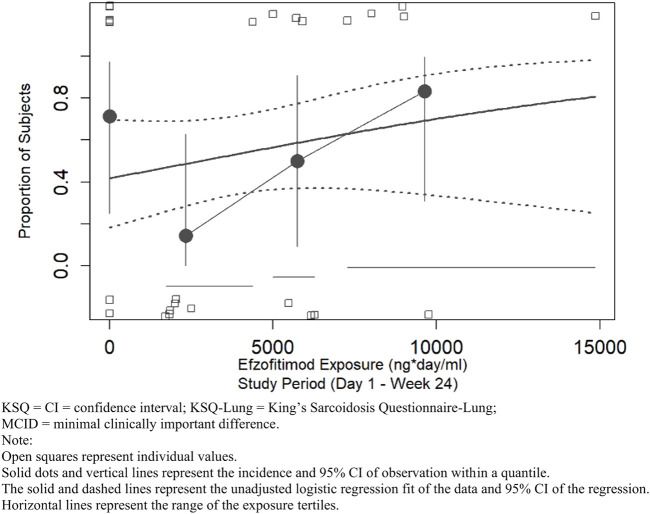
KSQ-lung score—proportion of participants achieving MCID threshold.

A linear logistic regression analysis was performed for KSQ-Lung score to explore the relationship between efzofitimod exposure and the probability of achieving an MCID in KSQ-Lung score. Responders in this analysis were defined as participants who achieved an increase of ≥4 points in KSQ-Lung score (Baughman et al., 2021b). As shown in Figure 8, an increase in the proportion of participants achieving the MCID was observed with increasing efzofitimod exposure. A clear placebo effect on this KSQ-Lung score was apparent, in that seven of the placebo participants achieved the MCID (as indicated by the open squares in Figure 8), resulting in a significant impact of the intercept in the final model.

### 3.4 Dose selection

**FIGURE 9:**
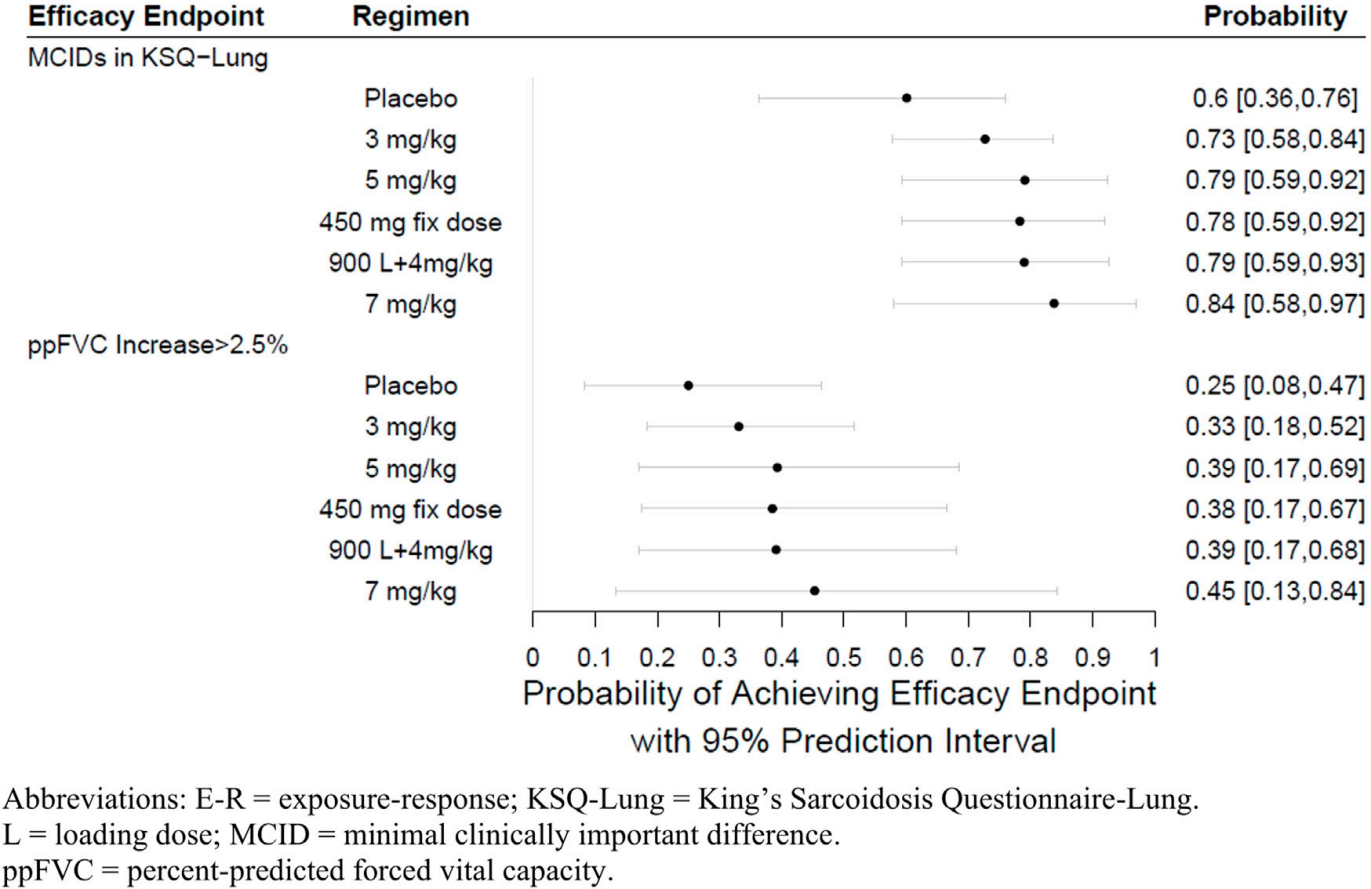
E-R simulation forest plots at week 24.

The final E-R logistic regression models developed for ppFVC and KSQ-Lung score responders were used to predict probability of response for dosing regimens not tested in the study (Section 2.6). The simulation results are shown in Figure 9. Based on the simulations, the proportion of responders predicted for ppFVC and KSQ-Lung score are comparable for 5 mg/kg and a fixed dose of 450 mg, supporting either dose regimen for future evaluation. The increase in the proportion of responders at the higher dose of 7 mg/kg compared to 5 mg/kg was 5% for KSQ-Lung score and 6% for ppFVC. Given the small number of participants on which the model was built, these estimates are associated with wide confidence intervals. There appeared to be no advantage to the use of a regimen including a loading dose.

## 4 Discussion

In this analysis, data from a Phase 1 healthy volunteer study with frequent (rich) sampling, and data from a Phase 1b/2a study in participants with PS with less frequent (sparse) sampling, were used to build a PPK model. This model was then used to generate a complete concentration-time profile for each patient with PS. The model was subsequently tested against the actual (sparse) PK dataset from these participants. Next, the AUC data generated by the PPK model was used to build an E-R model. The E-R model was then used to estimate the effect of efzofitimod exposure on three efficacy endpoints: mean daily OCS dose, ppFVC and KSQ-Lung score.

The concentration-time profile of efzofitimod was suggestive of a three-compartment model and the Vd was equivalent to the extracellular space. The three-compartment model and the Vd were consistent with that seen for other fusion proteins. In this model, CrCL was a significant covariate. This is consistent with other biologics with a molecular weight of <69 kDa. The molecular weight of efzofitimod is 64.5 kDa suggestive of a limited role for renal function in efzofitimod CL. Bilirubin was also a significant covariate. Some fusion proteins may be metabolized in the liver via proteolysis following endocytosis, however the hepatic elimination for fusion proteins is not as clinically relevant as it is for small molecules (Chen et al., 2012). Although body weight was positively correlated with both central and peripheral Vd, the effects were relatively small based on the PPK model. This supports the use of either a fixed dose or a weight-based dose for future studies.

An E-R analysis was undertaken as supportive evidence for efficacy and to provide PoC. An exposure (surrogate for dose)-response analysis is useful in early development to improve understanding of the safety and efficacy of a drug. Health authority guidance supports use of E-R information. For example, the US FDA has two guidances that recommend use of E-R analyses: the non-inferiority guidance (US FDA, 2016) and the E-R guidance (US FDA, 2003).

In early phase studies, the primary objective is safety and tolerability with efficacy being a secondary or exploratory objective. Such studies are rarely powered to demonstrate superiority of an active arm against placebo (Stallard, 2012). In an E-R analysis, response is the dependent parameter and exposure (concentration), the independent parameter. A single dose level will result in different concentrations in different patients. If a response is causally associated with a drug, then the response is typically related to the concentration of the drug in patients. It is this attribute of causation that is evaluated. Generally, it is recommended to perform E-R analyses on data from randomized controlled studies, as opposed to observational studies, as randomized controlled trials allow for the minimization of the impact of other factors when evaluating causality. E-R analysis is particularly useful where the number of patients with the disease of interest is small. This is typical for rare diseases or diseases in special populations like the pediatric or geriatric population. The US Food and Drug Administration’s (FDA) designation of efzofitimod as an orphan drug for the treatment of sarcoidosis is listed in the publicly available database (US FDA, 2022a). An E-R analysis for the Phase 1b/2a study is warranted as it is both early development and sarcoidosis is considered a rare disease.

The Phase 1b/2a study was not powered to demonstrate efficacy of one dose arm over another, or over placebo. The E-R analyses were not powered to show statistical significance. Confidence and prediction intervals have been included to convey uncertainty of point estimates (Amrhein et al., 2019). The consensus around the statistical methodology for E-R has been evolving over the last decade. The drug regulators in both the US and European Union have issued white papers on the statistical aspects of an E-R analysis (US FDA fit-for purpose (US FDA, 2022b), European Medicines Agency qualification (EMA, 2014)). When planning and performing E-R analyses to demonstrate PoC, a Type I error control may not be required (i.e., chance of making a false conclusion is no more than some prespecified value, α, typically 5%). Methods to control Type I error are available (e.g., multiple comparison procedure—modeling) in which all steps of model-building are prespecified. However, when Type I error control is less important (e.g., in early phase drug development), a more heuristics-based approach may be warranted. Such approaches enable a range of models and model assumptions to be evaluated on a more exploratory basis before determining the final model that fits the data best.

Given the early phase of development of efzofitimod, we performed an E-R analysis using the heuristics-based approach as supportive evidence for efficacy. Since the drug exposure is different between patients given the same (nominal) dose, E-R analysis offers more insight to the relationship with the response than an aggregate dose response analysis. We explored E-R relationships between endpoints as a continuous variable (percent change from baseline) and PK exposure and then fitted a linear regression model to the data.

A key finding in this analysis was that the slopes for all the end points showed an improving trend with exposure. Administration of efzofitimod led to an exposure-dependent decrease in the extent of OCS usage. Furthermore, given the responsiveness of FVC to oral steroids, it was interesting to note that even as steroids were tapered, increasing efzofitimod concentrations were associated with an increase in FVC. Likewise, the KSQ-Lung score appeared to improve with increased efzofitimod concentrations.

Dose selection for future studies of efzofitimod was performed using E-R logistic regression models developed for ppFVC and KSQ-Lung responder criteria. These models were used to predict probability of response for dosing regimens not tested in the study. Although an incremental benefit is predicted between 5 and 7 mg/kg, extrapolation beyond the range of exposures studied comes with an increasing level of uncertainty.

Overall, these preliminary findings of a positive E-R across multiple relevant endpoints support the claim that PoC has been established for the use of efzofitimod in PS. The potential for efzofitimod to have an SSE could also lead to decreases in steroid-related toxicities. The trends toward improved pulmonary function and quality of life make efzofitimod an attractive molecule for further clinical study. Efzofitimod doses of 3 and 5 mg/kg are being evaluated in a confirmatory Phase 3 study in PS (ClinicalTrials.gov, Identifier NCT05415137, 2022).

## 5 Conclusion

Sarcoidosis is a debilitating disease with few effective treatment options. The US FDA has not approved any new drugs for this disease since 1952 (prior to current health authority guidelines). The medical need for new treatments for sarcoidosis patients with pulmonary disease remains profound with 1 in 10 patients dying from the disease within 10 years.

These preliminary findings of a positive E-R across multiple efficacy endpoints support the claim that efzofitimod displays PoC in PS and is a fit candidate for a larger confirmatory Phase 3 study.

## Data Availability

The original contributions presented in the study are included in the article/Supplementary Materials. Further inquiries can be directed to the corresponding author.

## Abbreviations

AUC, area under the concentration time curve; CL, clearance; CrCL, creatinine clearance; E-R, exposure-response; ERS, European Respiratory Society; FDA, Food and Drug Administration; FVC, forced vital capacity; ILD, interstitial lung disease; KSQ-Lung, King’s Sarcoidosis Questionnaire-Lung; MCID, minimal clinically important difference; OCS, oral corticosteroids; PoC, proof of concept; PK, pharmacokinetic; ppFVC, percent-predicted forced vital capacity; PPK, population pharmacokinetic; PROs, patient-reported outcomes; PS, pulmonary sarcoidosis; SSE, steroid-sparing effect; US, United States; V1, central volume of distribution; V2, peripheral volume of distribution 2; V3, peripheral volume of distribution 3; Vd, volume(s) of distribution.
