# Supplementary material for: Exposure-response analyses of efzofitimod in patients with pulmonary sarcoidosis
Source: Front Pharmacol. 2023 Oct 3;14:1258236. doi: 10.3389/fphar.2023.1258236 (PMC10580085; doi:10.3389/fphar.2023.1258236)
Supplement: Supplementary file 1 [file DataSheet1.docx]

Supplementary MATERIAL

Files: Table S.1

Title: Parameter Estimates for the Final PPK Model

Description: PK parameter and covariates for the population pharmacokinetic model

Files: Equations

Title: Final linear logistic regression equations

Description: Logistic regression equations for exposure-response analysis

| Table S.1 Parameter Estimates for the Final PPK Model | | | | |
| --- | --- | --- | --- | --- |
| Parameter | Description | Unit | Estimate | %RSE |
| CL | Clearance | L/day | 1.68 | 2.93 |
| V1 | Central volume of distribution | L | 3.94 | 3.32 |
| Q2 | Inter-compartmental clearance 2 | L/day | 2.4 | 6.96 |
| V2 | Peripheral volume of distribution 2 | L | 3.3 | 17.92 |
| Q3 | Inter-compartmental clearance 3 | L/day | 1.2 | 13.76 |
| V3 | Peripheral volume of distribution 3 | L | 7.43 | 6.19 |
| 2  ω (CL) | Between-subject variance of CL | - | 0.03 | 45.98 |
| 2  ω (V1) | Between-subject variance of V1 | - | 0.01 | 28.11 |
| 2  ω (Q2) | Between-subject variance of Q2 | - | 0.06 | 44.05 |
| 2  ω (V2) | Between-subject variance of V2 | - | 0.1 | 36.1 |
| 2  ω (Q3) | Between-subject variance of Q3 | - | 0 FIX | - |
| 2  ω (V3) | Between-subject variance of V3 | - | 0.01 | 54.14 |
| BILI_CL | Power of bilirubin effect on CL | - | -0.17 | 23.02 |
| CRCL_CL | Power of CrCL effect on CL | - | 0.71 | 20.87 |
| BWT_V1 | Power of body weight on V1 | - | 0.77 | 20.11 |
| BWT_V2 | Power of body weight on V2 | - | 1.52 | 31.87 |
| 2  ε (Phase 1) | Log-additive residual variance of the Phase 1 study | - | 0.01 | 17.94 |
| 2  ε (Phase 2) | Log-additive residual variance of the Phase 2 study | - | 1.63 | 7.48 |
| HL | Half-life accounting for all phases of elimination | day | 11.30 | - |
| Terminal HL | Terminal-phase half-life | day | 9.00 | - |
| Abbreviations: %RSE=percent relative standard error (SE/estimate × 100%); CrCL = creatinine clearance; PPK=population pharmacokinetic; ω2=variance of between-subject variability for that parameter; ε2=variance of log-additive within-subject variability | | | | |

Equations: Final linear logistic regression equations

**Eq. A.1: Mean Daily OCS Dose Post-Taper Period - Percent Change from Baseline (supporting Figure 4)**

$$drug-adjusted AUC percent change from baseline = -0.001731 \times AUC\left( ng\times\frac{day}{mL} \right)-43.01$$

**Eq. A.2: ppFVC at Week 24 - Percent Change from Baseline (supporting Figure 5)**

$$ppFVC percent change from baseline = 0.0005061\times AUC\left( ng\times\frac{day}{mL} \right)+1.182\times baseline ppFVC-17.15$$

**Eq. A.3: KSQ-Lung Score at Week 24 - Percent Change from Baseline (supporting Figure 6)**

$$KSQ-Lung score percent change from baseline=0.001935\times AUC\left( ng\times\frac{day}{mL} \right)+50.1$$

**Eq. A.4: ppFVC – Proportion of Participants Achieving MCID Threshold, (supporting Figure 7)**

$$\log it\left( \Pr obability \right)=-10.25+ 0.0000953 \times AUC\left( ng\times\frac{day}{mL} \right)+0.11 \times Baseline ppFVC \left( \% \right)$$

**Eq. A.5: KSQ-Lung score - Proportion of Participants Achieving MCID Threshold, (supporting Figure 8)**

$$\log it\left( \Pr obability \right)=6.38+ 0.000219 \times AUC\left( ng\times\frac{day}{mL} \right)-0.15 \times Baseline KSQL Score \left( point \right)$$
